# The Disruptive Force of Real-World Evidence

**DOI:** 10.3390/jcm12124026

**Published:** 2023-06-13

**Authors:** Marcus Schmitt-Egenolf

**Affiliations:** 1 Department of Public Health and Clinical Medicine, Dermatology, Umeå University, SE-901 85 Umeå, Sweden; marcus.schmitt-egenolf@umu.se; 2 Centre for Pharmacoepidemiology, Karolinska Institutet, SE-171 77 Stockholm, Sweden

## 1. Effectiveness and Efficacy

Evidence-based medicine was in the past primarily based on the (meta-)analysis of randomized clinical trials (RCTs). However, it has been shown repeatedly that RCT populations and settings do not represent real-world settings. Study protocol requirements chosen by the sponsor create an artificial situation that can be quite remote from real life [1]. Consequently, the concepts ‘effectiveness’ and ‘efficacy’ have been established to differentiate the potentially artificial picture provided by RCTs compared to what we see in real life. The results from real-world evidence (RWE) studies (such as those obtained from registries, claim databases, health surveys or electronic medical records) typically include larger unselected sample sizes with longer follow-up periods of the real-world population. However, due to the common lack of randomization in RWE studies, we need to keep in mind the potential for bias and confounding.

RCT participants may differ from most patients seen in clinical practice with respect to concomitant diseases, medications, and compliance as well as sex and age distribution [2]. In addition, the duration of RCTs is in general much shorter than the expected time of usage of the investigated drug. For example, to follow-up patients with a chronic disease such as asthma for at least one year, as in the recent study by Oishi et al. [3], in a real-world study of clinical and deep remission in response to biologics should be a standard. However, RCTs regularly have shorter follow-up periods. Consequently, RCT-based conclusions can be true for the RCT-population (high internal validity), but may at the same time be irrelevant to the real-world setting (low external validity).

**Figure 1:**
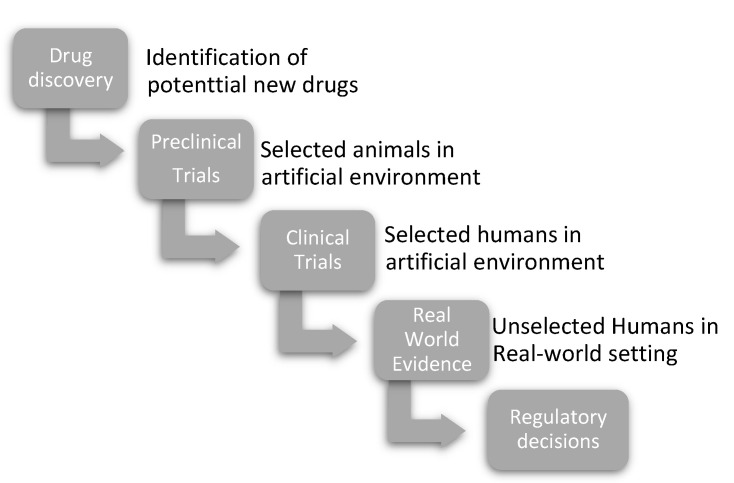
The stepwise approach in the evaluation of therapies. In the stepwise evaluation of therapies, every step is informed by the previous step. Consequently, as real-world evidence has become readily available, it has become more infrequent that regulatory decisions are made based only on RCTs.

One could hope that this criticism of the unrepresentativeness of RCTs might have led to a change for the better. This appears, unfortunately, not to be the case. For example, Asai et al. [4] recently analyzed to what extent real-world patients with infective endocarditis would be included in RCTs. Only every fourth patient (26%) fulfilled the eligibility criteria for RCTs. Patients in the “RCT appropriate group” were younger, had fewer comorbidities, milder disease severity, and significantly longer overall survival times than those in the “RCT inappropriate group”. It is evident that this misrepresentation of the real world in RCTs can lead to problems on many levels. RCTs should consequently be used as one of several stepping stones in the evaluation of therapies before finally acquiring RWE (Figure 1), while only the latter can be used as decisive evidence for both reimbursement and safety. However, as it takes time to collect RWE, preliminary decisions may be based on RCTs alone.

## 2. RCTs and RWE Can Complement Each Other

A recent example of how RWE can complement earlier published RCTs is the investigation of effectiveness of dupilumab and upadacitinib in the treatment of atopic dermatitis by Kiefer et al. [5] in a small real-world cohort. As a head-to-head comparison of drugs in the same generation is rarely performed in RCTs due to a lack of motivation from the sponsor’s side, this example shows an additional benefit of the RWE approach. Both drugs significantly improved, to a similar extent, the severity of eczema and itching and the health-related quality of life. Additionally, when investigating safety, RCTs and RWE can complement each other. This has recently been exemplified by Yao et al. [6], who found that including RWE in a rare events meta-analysis had the potential to corroborate findings from RCTs, increase precision, and consequently enhance the decision-making process.

An interesting emerging field is the application of artificial intelligence in the form of machine learning to the analysis of real-world data, as recently performed for the prediction of potential features increasing the risk of developing long COVID by Kessler et al. [7]. RWE data are unlike RCT data in that they are often not obtained in a protocol-driven way and can be difficult to analyze with traditional statistical methods due to their structural diversity. Therefore, RWE data can profit particularly from artificial intelligence-supported data analysis.

## 3. Conclusions

The disruptive force of RWE as a reality check of knowledge derived from RCTs for medical decision-making has become generally recognized and will probably continue to develop in the future.
